# Low Genetic Diversity in *Melanaphis sacchari* Aphid Populations at the Worldwide Scale

**DOI:** 10.1371/journal.pone.0106067

**Published:** 2014-08-22

**Authors:** Samuel Nibouche, Benjamin Fartek, Stelly Mississipi, Hélène Delatte, Bernard Reynaud, Laurent Costet

**Affiliations:** 1 Cirad, UMR PVBMT, Saint-Pierre, La Réunion, France; 2 Université de la Réunion, UMR PVBMT, Saint-Pierre, La Réunion, France; Emory University, United States of America

## Abstract

Numerous studies have examined the genetic diversity and genetic structure of invading species, with contrasting results concerning the relative roles of genetic diversity and phenotypic plasticity in the success of introduced populations. Increasing evidence shows that asexual lineages of aphids are able to occupy a wide geographical and ecological range of habitats despite low genetic diversity. The anholocyclic aphid *Melanaphis sacchari* is a pest of sugarcane and sorghum which originated in the old world, was introduced into the Americas, and is now distributed worldwide. Our purpose was to assess the genetic diversity and structuring of populations of this species according to host and locality. We used 10 microsatellite markers to genotype 1333 individuals (57 samples, 42 localities, 15 countries) collected mainly on sugarcane or sorghum. Five multilocus lineages (MLL) were defined, grouping multilocus genotypes (MLG) differing by only a few mutations or scoring errors. Analysis of a 658 bp sequence of mitochondrial COI gene on 96 individuals revealed five haplotypes, with a mean divergence of only 0.19 %. The distribution of MLL appeared to be strongly influenced by geography but not by host plant. Each of the five MLL grouped individuals from (A) Africa, (B) Australia, (C) South America, the Caribbean and the Indian Ocean including East Africa, (D) USA, and (E) China. The MLL A and C, with a wide geographic distribution, matched the definition of superclone. Among aphids, *M. sacchari* has one of the lowest known rates of genetic diversity for such a wide geographical distribution.

## Introduction

Range expansion of exotic species can result from either evolutionary adaptation or generalism and plasticity often associated with a change in niche [1]. Genetic diversity is required for evolutionary adaptation, but a reduction in genetic diversity in invasive populations compared to populations in their native range is expected and often observed [2]–[5]. Organisms with clonal reproduction may exhibit reduced genetic diversity within populations, as better-adapted clonal genotypes expand and dominate available resources [6]. In aphids, the concept of “superclones” emerged [7] when a few asexual genotypes of the same species were able to colonize a wide geographical or ecological range of habitats [8]–[11]. The capacity of these populations to adapt to different conditions could be the result of a preadaptation capacity for phenotypic plasticity rather than local selection acting on genetic diversity [12], [13]. What is more, this capacity may be enhanced by their high rate of reproduction and population expansion [14]. For these reasons, clonal aphids are good models to assess the ability of asexual lineages to show rapid and widespread adaptive changes to ecological conditions [15].

The old world genus *Melanaphis* van der Groot 1917 comprises around 20 species mainly associated with Poaceae, most of which originate from East Asia [16]. The sugarcane aphid *Melanaphis sacchari* (Zehtner, 1897) (Homoptera, Aphididae), which is considered to be mainly anholocyclic, is present in America, Australia, Asia and Africa. *M. sacchari* is known to be invasive in continental US [17], [18] and in Central and South America [16]. The host range of this species is restricted to Poaceae [2], [19]. Blackman et al. [20] hypothesised that *Melanaphis* individuals originating from sorghum or sugarcane were distinct taxa, referred to as *M. sorghi* and *M. sacchari* respectively, even though their host plant preference was not absolute. In their catalogue, Remaudière and Remaudière [21] considered *M. sorghi* to be a synonym for *M. sacchari*, but both forms were still listed as separate taxa by Blackman and Eastop [22], and it is still not clear whether *M. sacchari* constitutes a single species or a complex of sibling taxa.

In any case, *M. sacchari* is a major pest of sorghum and sugarcane. On sugarcane, it is considered to be the most common and most efficient vector of the *Sugarcane yellow leaf virus* (ScYLV), which causes yellow leaf disease [23], [24], a disease of worldwide economic importance [25]–[27]. The aphid is also a major pest of sorghum, causing direct damage (sap feeding) and indirect damage (sooty mould) [19]. Varietal resistance against *M. sacchari* is one of the main control tactics suggested both for sugarcane [28], [29] and sorghum [19], [30]. Most plant resistance to aphids is specific to a single aphid species or to a few biotypes within a species [31] and it has been demonstrated that variability exists among clonal lineages of aphids in their response to resistant cultivars [32], [33]. Therefore, characterisation of the genetic diversity of aphids is critical for breeding durable and efficient resistance, which has to account for the worldwide diversity of these pests and the potential emergence of new invasive biotypes.

Based on a worldwide sample of aphids covering its area of distribution (home range as well as invasive range) and using microsatellite markers and sequencing of a fragment of the mitochondrial cytochrome c oxidase I gene (COI), the purpose of this study was to evaluate 1) the mode of reproduction of *M. sacchari*; 2) molecular evidence for the existence of sibling species; and 3) its genetic diversity and structuring according to host and locality.

## Materials and Methods

### Insect samples

Here, an ‘individual’ refers to one individual aphid and a ‘sample’ refers a several individuals collected from the same host plant species in a given locality and date. The complete set of individuals (Table S1) comprised 57 samples from 42 localities in a total of 15 countries or provinces, and from five host plants: sugarcane, pearl millet, and three wild or cultivated sorghum species (*Sorghum bicolor*, *S. halepense, S. verticilliflorum*). The three sorghum species were considered as a single host plant, hereafter named ‘sorghum’.

Aphids were collected from wild or cultivated plants and placed in 70% ethanol in Eppendorf tubes, kept frozen at −80°C until they were processed. Only a few aphids were collected on each plant sampled to avoid collecting several individuals from the same colony.

Sampling was carried out from 2002 to 2009 by our team in Reunion Island and by colleagues in the other parts of the world (see acknowledgements). Geographic coordinates of sampling localities are provided in Table S1. No specific permissions were required for sampling aphids in these locations. The field studies did not involve endangered or protected species.

### DNA extraction, genotyping and sequencing

#### DNA extraction

DNA was extracted using the “salting-out” protocol of Sunnucks and Hales [34]. Briefly, it consists in extracting DNA from whole aphids by crushing them in a TNES/Proteinase K buffer and precipitating DNA in ethanol. This method is simple and fast, and provided sufficient DNA for phylogeny and microsatellite PCR analyses.

#### Genotyping

According to their polymorphism, ten microsatellite loci (Tab. 1) were selected among the 14 previously developed by our team for *M. sacchari* [35]. PCR reactions were performed with labelled primers and multiplexed into two mixes (Type-it, standard procedure, Qiagen), and the following thermocycling protocol was used: denaturation at 95°C for 15 min, 25 denaturation cycles for 30 s at 94°C, a 1-min 30 s annealing step at 54°C, and a 30-s elongation step at 72°C. We used an ABI prism 3110 for genotyping after addition of an internal size standard for each sample (GeneScan LIZ 500, Applied Biosystems). Alleles were identified at each locus by comparison with the size standard using GeneMapper version 2.5 software (Applied Biosystems).

**Table 1:** Observed microsatellite Multi Locus Genotypes (MLG): allele size (bp) at each locus, distribution by host plant and in each of the five Multi Locus Lineages (MLL) defined with GENCLONE. Within MLL allelic variations are in bold.

MLL	MLG	CIR-Ms-G08	CIR-Ms-G403	CIR-Ms-B03	CIR-Ms-C08	CIR-Ms-G01	CIR-Ms-E01	CIR-Ms-G12	CIR-Ms-E03	CIR-Ms-D02	CIR-Ms-G02
MLL A	Ms5	229/229	253/253	215/215	189/203	206/**210**	245/247	214/216	176/176	220/**228**	**250**/**316**
	Ms30	229/229	253/253	215/215	189/203	**204**/206	245/247	214/216	176/176	220/**228**	**250**/**318**
	Ms31	229/229	253/253	215/215	189/203	206/**206**	245/247	214/216	176/176	228/**230**	**254**/**314**
	Ms41	229/229	253/253	215/215	189/203	206/**206**	245/247	214/216	176/176	220/**226**	**252**/**314**
	Ms42	229/229	253/253	215/215	189/203	206/**206**	245/247	214/216	176/176	220/**226**	**254**/**314**
	Ms301	229/229	253/253	215/215	189/203	206/**206**	245/247	214/216	176/176	220/**228**	**248**/**312**
	Ms302	229/229	253/253	215/215	189/203	206/**206**	245/247	214/216	176/176	220/**228**	**248**/**314**
	Ms303	229/229	253/253	215/215	189/203	206/**206**	245/247	214/216	176/176	220/**228**	**250**/**298**
	Ms304	229/229	253/253	215/215	189/203	206/**206**	245/247	214/216	176/176	220/**228**	**250**/**310**
	Ms305	229/229	253/253	215/215	189/203	206/**206**	245/247	214/216	176/176	220/**228**	**250**/**312**
	Ms306	229/229	253/253	215/215	189/203	206/**206**	245/247	214/216	176/176	220/**228**	**250**/**314**
	Ms307	229/229	253/253	215/215	189/203	206/**206**	245/247	214/216	176/176	220/**228**	**250**/**316**
	Ms308	229/229	253/253	215/215	189/203	206/**206**	245/247	214//216	176/176	220/**228**	**250**/**318**
	Ms309	229/229	253//253	215/215	189//203	206/**206**	245/247	214/216	176/176	220/**228**	**250**/**343**
	Ms310	229/229	253/253	215/215	189/203	206/**206**	245/247	214/216	176/176	220/**228**	**250**/**345**
	Ms311	229/229	253/253	215/215	189/203	206/**206**	245/247	214/216	176/176	220/**228**	**252**/**312**
	Ms312	229/229	253/253	215/215	189/203	206/**206**	245/247	214/216	176/176	220/**228**	**252**/**314**
	Ms313	229/229	253/253	215/215	189/203	206/**206**	245/247	214/216	176/176	220/**228**	**254**/**312**
	Ms314	229/229	253/253	215/215	189/203	206/**206**	245/247	214/216	176/176	220/**228**	**254/314**
	Ms315	229/229	253/253	215/215	189/203	206/**206**	245/247	214/216	176/176	220/**228**	**256/314**
MLL B	Ms1	233/233	253/259	213/215	197/199	185/206	247/247	212/216	188/**193**	226/**232**	199/**199**
	Ms2	233/233	253/259	213/215	197/199	185/206	247/247	212/216	188/**193**	226/**252**	199/**199**
	Ms121	233/233	253/259	213/215	197/199	185/206	247/247	212/216	188/**191**	226/**232**	199/**199**
	Ms122	233/233	253/259	213/215	197/199	185/206	247/247	212/216	188/**191**	226/**232**	199/**201**
MLL C	Ms6	233/233	251/259	213/213	197/**199**	185/**210**	247/247	212/216	186/193	228/**234**	199/**199**
	Ms7	233/233	251/259	213/213	197/**199**	185/**210**	247/247	204/212	186/193	228/**232**	199/**199**
	Ms8	233/233	251/259	213/213	197/**199**	185/**212**	247/247	212/216	186/193	228/**232**	199/**199**
	Ms11	233/233	251/259	213/213	197/**199**	185/**210**	247/247	212/216	186/193	228/**232**	199/**199**
	Ms12	233/233	251/259	213/213	**195/**197	185/**210**	247/247	212/216	186/193	228/**232**	199/**205**
	Ms15	233/233	251/259	213/213	197/**199**	185/**210**	247/247	212/216	186/193	228/**232**	199/**201**
	Ms16	233/233	251/259	213/213	**195/**197	185/**210**	247/247	212/216	186/193	228/**232**	199/**203**
	Ms21	233/233	251/259	213/213	**195/**197	185/**210**	247/247	212/216	186/193	228/**234**	199/**203**
MLL D	Ms9	233/233	251/259	213/213	197/199	185/206	247/247	212/**216**	186/188	226/234	201/201
	Ms10	233/233	251/259	213/213	197/199	185/206	247/247	212/**218**	186/188	226/234	201/201
MLL E	Ms32	229/229	253/253	215/215	197/197	198/206	245/245	208/212	**216/**224	220/222	216/216
	Ms33	229/229	253/253	215/215	197/197	198/206	245/245	208/212	**224**/224	220/222	216/216

#### Sequencing

A total of 91 aphids were chosen among the worldwide sample to represent different combinations of region and host plant. COI fragments were amplified using the LCO1490 and HCO2198 primers designed by Folmer et al. [36]. PCR was carried out using the protocol of Kim and Lee [37]. PCR products were purified and sequenced by a subcontractor (Cogenics), and a consensus sequence of 658 pb was chosen for later analyses.

### Data analysis

#### Clonal diversity analysis

Micro-Checker software [38] was run on the whole population. No evidence was found for the presence of null alleles. Any single combination of alleles was retrieved from genotyping data and arranged as unique multilocus genotypes (MLGs). Given the clonal reproduction of *M. sacchari*, we assumed that the different occurrences of the same MLG in a sample were the result of local clonal reproduction. We therefore retained a single representative of each MLG in each of the 57 samples for genetic and diversity analysis.

Using GENCLONE software [39], we computed a matrix of pairwise genetic distance between MLGs computed as the number of allelic differences between MLGs [40]. Examination of the distribution of these distances enabled us to define a threshold below which MLGs were considered to belong to the same multilocus lineage (MLL), i.e. genotypes which differed slightly due to mutation or scoring errors according to Arnaud-Haond et al. [40]. The same matrix of pairwise distances was also used to construct a minimum spanning network using HAPSTAR software [41]. On the set of identical loci within each MLL, we computed p_sex_, the probability that the repeated MLGs originated from distinct sexual reproductive events. A p_sex_ value lower than 0.01 supported the hypothesis that MLGs originated from the same MLL [40]. To describe clonal diversity, we computed the clonal richness index as R  =  (G-1)/(N-1), where G is the number of genotypes detected (either MLGs for R_MLG_ or MLL for R_MLL_), and N is the number of samples [42].

#### Phylogenetic analysis

Sequence alignments of the COI gene were performed using Geneious software version 5.6.6 [43]. Five sequences from *M. sacchari* individuals collected in India [44] were retrieved from GenBank and added to our data. Four sequences from three species of the *Melanaphis* genus were also retrieved from GenBank and used as outgroups: *M. donacis* (referenced HQ443314), *M. bambusae* (referenced EU701747 and EU701746) and *M. japonica* (referenced GU457792). Maximum Likelihood inference performed with MEGA6 [45] was used to choose the most reliable evolutionary model of base substitution to infer the evolutionary history. Based on the AICc criterion, the best model proved to be the General Time Reversible model with gamma distribution of evolutionary rates among sites (GTR+G) [46]. The GTR+G model was then used with MEGA6 to reconstruct the subsequent phylogenetic tree through the Maximum Likelihood method, with 10,000 bootstrap replicates for branch support.

#### Population genetic analysis

We used GENEPOP [47] to compute population genetics parameters for each of the MLLs delimited by GENCLONE. We tested departures from Hardy–Weinberg equilibrium and heterozygote deficit and excess, and calculated population fixation index values (F_is_). Genetic differentiation between MLLs was tested with a G test and pairwise F_ST_ were computed.

## Results

### Genetic and clonal diversity

We genotyped a total of 1333 aphids using the ten microsatellite markers. When we retained a single representative of each MLG in each of the 57 samples, this yielded a dataset containing 98 individuals.

**Figure 1:**
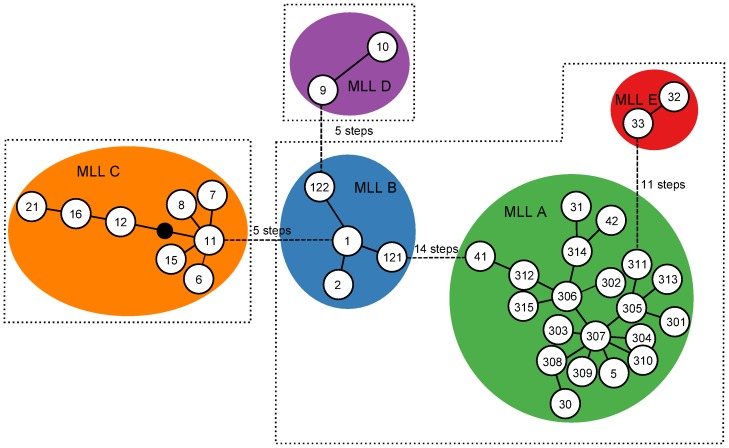
Minimum spanning network of *Melanaphis sacchari* microsatellite distances computed as the number of allele differences between MLGs. Each node represents one step in the network, i.e. a distance of one allele. The numbers in the circles represent MLGs according to Table 1. Coloured backgrounds represent the Multi Locus Lineages (MLLs). MLGs in the same dashed line box share the same COI haplotype.

Global genetic diversity was low, with 36 MLGs found (Table 1). Global clonal richness was also low, with a R_MLG_  = 0.361. The distribution of the pairwise number of different alleles between MLG appeared multimodal, with a first minimum located at a distance of five alleles (Figure S1). Grouping MLGs which differed by one to four alleles defined five groups. Calculation of p_sex_ on the set of identical loci within each of these five groups yielded values <0.01, confirming that the MLGs within each group were unlikely to have derived from distinct reproductive events. We therefore considered that the five groups defined five multilocus lineages (MLLs) which grouped slightly distinct MLGs resulting from step mutations or scoring errors (Table 1, Figure 1). Considering the five MLLs, clonal richness was very low, as shown by the R_MLL_  = 0.041.

### Phylogenetic relationship within samples

**Figure 2:**
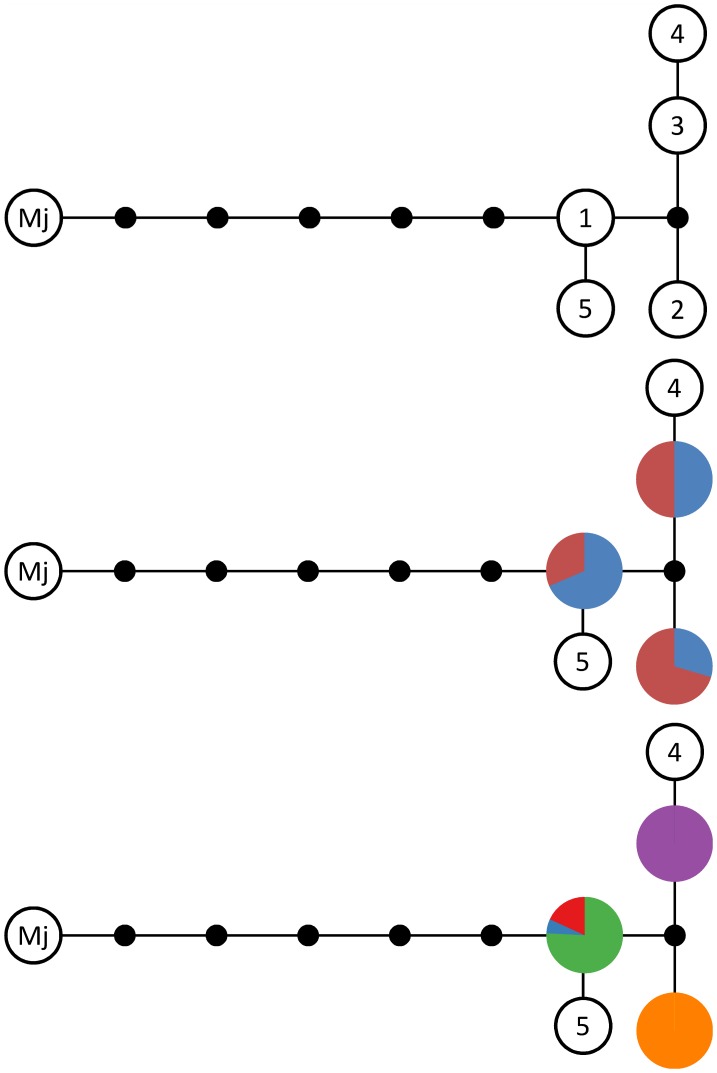
COI haplotype network (top), in which *Melanaphis sacchari* COI sequences originating from the present study are numbered from 1 to 3. *M. sacchari* GenBank COI sequences from India [44] are numbered 4 (JX051388, JX05189, JX051390) and 5 (HQ112185, JX051402). Mj  =  *Melanaphis japonica* COI sequence from GenBank (GU457792). Distribution as a function of host plant (middle): sorghum (blue) vs. sugarcane (red). Distribution as a function of MLL (bottom): A (green), B (blue), C (yellow), D (violin), E (red).

Within our 91 *M. sacchari* COI sequences, only three distinct haplotypes were observed (Figure 2). One haplotype was observed in individuals belonging to MLL-A, MLL-B or MLL-E (Figure 1, Figure 2). The second haplotype was only observed in individuals belonging to MLL-C. The third haplotype was observed in individuals belonging to MLL-D. No association of haplotypes with the host plant was observed (Figure 2). These three haplotypes differed from the two available in GenBank from five Indian samples, giving a total of five haplotypes and five nucleotide substitutions among 96 *M. sacchari* individuals. The phylogenetic tree built from a 658 bp fragment of the COI gene clearly separated the four *Melanaphis* species with >80% bootstrap support (Figure S2). But within the *M. sacchari* sequences, the presence of distinct taxa was not supported by bootstrap analysis at the 80% threshold. Intraspecific genetic divergence in *M. sacchari* was low, with a mean pairwise divergence of 0.19% (range 0.000.61%). When the five *M. sacchari* sequences retrieved from GenBank were excluded, the mean divergence was 0.17% (range 0.000.30%). The mean divergence of *M. sacchari* sequences with the closest taxa, *M. japonica*, was 1.06% (range 0.92%1.39%).

### Standard population genetics

**Figure 3:**
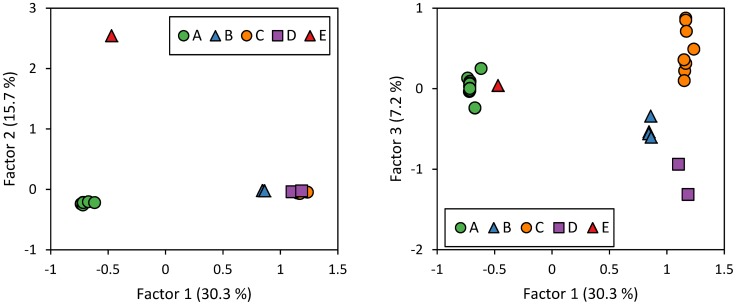
Factorial correspondence analysis of microsatellite data with GENETIX. Each symbol represents one of the 36 MLGs. Colours and letters refer to Multilocus Lineage (MLL) assignment with GENCLONE.

Plotting the results of the factorial correspondence analysis with GENETIX confirmed the grouping of the 36 MLGs in five MLLs (Figure 3). Factor 1 distinguished MLL-A and MLL-E, and a group formed by the three MLL-B, MLL-C and MLL-D. Factor 2 distinguished MLL-E from other MLLs. Factor 3 distinguished between MLL-B, MLL-C and MLL-D.

Genetic differentiation between the five MLLs was strong, with a highly significant F_st_ ranging from 0.262 to 0.694 (Table S2).

The five populations comprised by each distinct MLL differed significantly from Hardy-Weinberg equilibrium, and showed a clear signature of asexual reproduction with a significant heterozygote excess and negative F_IS_ values (Table S3).

### Geographical and host distribution of MLLs

**Figure 4:**
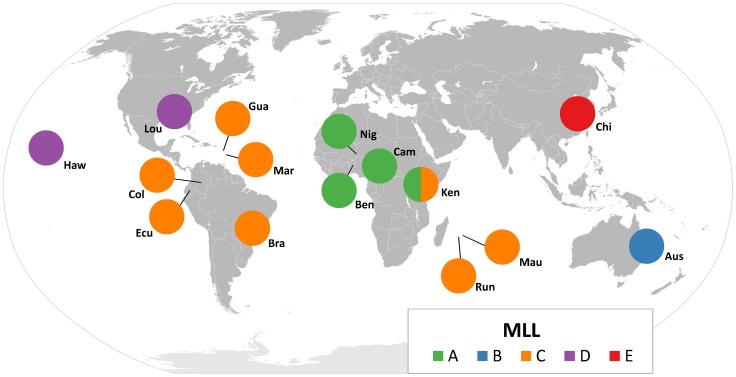
Relative geographical within-state distribution of Multilocus lineages (MLL). The size of circle is not proportional to the size of the sample. Aus  =  Australia, Bra  =  Brazil, Col  =  Columbia, Ecu  =  Ecuador, Gua  =  Guadeloupe, Haw  =  Hawaii, Lou  =  Louisiana, Mar  =  Martinique, Mau  =  Mauritius, Run  =  Reunion Island, Ben  =  Benin, Cam  =  Cameroon, Nig  =  Niger, Chi  =  China, Ken  =  Kenya.

Distribution of the MLLs revealed strong geographical structuring (Figure 4). MLL-A was observed in Africa, MLL-B was restricted to Australia, MLL-C exhibited the widest distribution area (South America, the Caribbean, the Indian Ocean and East Africa), MLL-D was observed in the USA, and MLL-E was only observed in China. Kenya was the only country where two MLLs were observed simultaneously: one sample (Ken1) contained a mix of MLL-A and MLL-B, one sample (Ken5) contained MLL-A alone, and three samples (Ken2, Ken3, Ken4) contained MLL-B alone.

No host plant structuring was observed: in all the countries where both sorghum and sugarcane samples were collected, each MLL was found on both host plants (Table 2).

**Table 2:** Distribution of the 98 individuals, a single representative of each MLG in each of the 57 samples, as a function of country/province and host plant.

State	Host plant	Number of individuals					Number of samples
		MLL-A	MLL-B	MLL-C	MLL-D	MLL-E	
Benin	sorghum	3					2
	sugarcane	6					1
	pearl millet	1					1
Cameroon	sorghum	20					5
Kenya	sorghum	2					1
	sugarcane	2		4			4
Niger	sorghum	5					2
Australia	sugarcane		11				7
Brazil	sugarcane			2			1
Columbia	sugarcane			7			3
Ecuador	sugarcane			1			1
Guadeloupe	sugarcane			6			5
Martinique	sugarcane			3			3
Mauritius	sugarcane			1			1
Reunion	sorghum			3			3
	sugarcane			7			5
Hawaii	sugarcane				3		3
Louisiana	sorghum				1		1
	sugarcane				8		7
China	sorghum					2	1

## Discussion

Molecular analysis revealed a very low genetic diversity among 57 samples collected in 15 countries on two main hosts, with 36 MLGs structured in five MLLs. The distribution of MLLs was strongly structured by geography but not by the host plant (sorghum vs. sugarcane).

Sequencing the COI ‘barcoding’ region, a typical locus used for species discrimination and phylogeny, particularly in aphids [48], [49], did not enable the detection of cryptic species in our samples. Specifically, we observed no molecular evidence for a clear separation into two species, *M. sacchari* and *M. sorghi*. We found sequence variations peaking at 0.61%, with a mean value of 0.19%, both of which are within the range of intraspecific divergence observed in the Aphididae family by Footit et al. [50] or Lee et al. [51].

### Reproduction

Population genetic parameters were consistent with populations which only reproduce by apomictic parthenogenesis, as previously described by Blackman and Eastop [22]. Each population significantly differed from Hardy-Weinberg equilibrium, with a high heterozygote excess. These features are a common consequence of populations which have reproduced clonally for a long time. In a global study of genetic diversity on *Aphis gossypii,* Carletto et al. [52] obtained similar results to ours as they observed low genetic diversity, with the predominance of a few clones at the worldwide scale reproducing only by apomictic parthenogenesis. But later, evidence for sexual reproduction of *A. gossypii* was found in Iran [53]. High genetic diversity and evidence for sexual reproduction was also observed in *A. gossypii* alate spring migrants in France [54]. Similarly, in the *Brachycaudus helichrysi* (Kaltenbach) sibling species H2, a sexually reproducing population was identified in India, despite almost exclusively clonal reproduction at the worldwide scale [11]. This shows that sexual admixture can still exist in a local population even in species which are highly clonal at the worldwide scale. A holocycle has been observed in *M. sacchari* in Asia [16] and this suggests that higher genetic diversity may exist in some parts of its geographic distribution area even if our sampling did not allow us to observe it.

### Geographic genetic structure

Microsatellite analyses showed that population structuring at the worldwide scale was only influenced by geography, delimiting five MLLs corresponding to five geographic zones: Africa, China, Australia, USA, and South America – Indian Ocean (including Kenya) and the Caribbean. Variation within each of the four biggest zones (excluding China where only one sample was analysed) was low, with MLGs within a zone differing by a few step mutations, which we suggest is due to one or few separate introductions. These results suggest that each of the five zones was colonized separately following the introduction of one or a few clones from the region of origin of *M. sacchari*, which the present study did not allow us to identify. At least two of the five MLLs covered a very wide geographic area and matched the pattern of a single asexual genotype with a high capacity for dispersal which would have spread across a large area: MLL-A was observed in West and East Africa, and MLL-C was observed in South America, the Indian Ocean, East Africa, and the Caribbean. Both MLLs match the definition of “superclone” [7], characterised by geographically and ecologically widespread distribution, which has already been documented in several aphid species [9], [10], [11], [55], [56].

The low rate of genetic diversity observed in the whole geographic area covered by our study, and the lack of published data about the dates of introduction of *M. sacchari* in the countries sampled, meant we were not able to reconstruct the invasion routes of this species. The only exception was continental USA. *M. sacchari* was first described in Hawaii in the late 19^th^ century [57], [58], and was first recorded in continental USA at the end of the 1970s in Florida [59], [60] and in 2001 in Louisiana [18]. In our study, almost all individuals sampled in Louisiana and Hawaii belonged to the same MLG, Ms9, and shared the same COI haplotype, neither of which were observed in other regions. This strongly suggests that *M. sacchari* was introduced in continental USA from Hawaii. This finding is noteable, as one would expect an introduction into continental USA from either South America or the Caribbean, a shorter invasion route. However, Mondor et al. [61] emphasized that the relationship between the colonization of the Hawaiian Islands by an aphid species and its presence in continental USA was due to the high rate of commercial exchanges between the two. Here we provide an example of reverse colonization from Hawaii to continental USA. In Kenya, MLL-C was observed in three samples from the coastal region of Kenya but was not found in a sample collected inland. This underlines the fact that, even though *M. sacchari* has been recorded in almost all areas where sugarcane is cultivated, the possibility for the expansion of some genotypes should be taken seriously, mainly its unknown potential impact on the epidemiology of the viral diseases it transmits.

## Supporting Information

Figure S1 **Distribution of the pairwise number of different alleles between MLGs.**
(PDF)

Figure S2 **Molecular Phylogenetic analysis by Maximum Likelihood method with bootstrap support (10,000 replicates) using 658 bp cytochrome c oxidase subunit I sequences from 100** ***Melanaphis*** **spp. individuals.**
(PDF)

Table S1 **Voucher number, sampling information, GenBank accession and SSR genotyping of individual aphids.**
(XLSX)

Table S2 **Genetic differentiation between MLLs: pairwise F_ST_ and significance of the G test computed with GENEPOP.**
(PDF)

Table S3 **Population genetics parameters for each MLL.**
(PDF)

## References

[1] Lee CE (2002) Evolutionary genetics of invasive species. Trends in Ecology & Evolution 17: 386–391.

[2] Amsellem L, Noyer J, Le Bourgeois T, Hossaert-McKey M (2000) Comparison of genetic diversity of the invasive weed *Rubus alceifolius* Poir. (Rosaceae) in its native range and in areas of introduction, using amplified fragment length polymorphism (AFLP) markers. Molecular Ecology 9: 443–455. 10736047 10.1046/j.1365-294x.2000.00876.x

[3] Bossdorf O, Auge H, Lafuma L, Rogers WE, Siemann E, et al (2005) Phenotypic and genetic differentiation between native and introduced plant populations. Oecologia 144: 1–11. 15891837 10.1007/s00442-005-0070-z

[4] Dlugosch K, Parker I (2008) Founding events in species invasions: genetic variation, adaptive evolution, and the role of multiple introductions. Molecular Ecology 17: 431–449. 17908213 10.1111/j.1365-294X.2007.03538.x

[5] Grapputo A, Boman S, Lindstroem L, Lyytinen A, Mappes J (2005) The voyage of an invasive species across continents: genetic diversity of North American and European Colorado potato beetle populations. Molecular Ecology 14: 4207–4219. 16313587 10.1111/j.1365-294X.2005.02740.x

[6] Vrijenhoek RC, Parker ED Jr (2009) Geographical parthenogenesis: general purpose genotypes and frozen niche variation. In: Schön I, Martens K, van Dijk P, editors. Lost sex. The evolutionary biology of parthenogenesis: Springer Netherlands. 99–131.

[7] Vorburger C, Lancaster M, Sunnucks P (2003) Environmentally related patterns of reproductive modes in the aphid *Myzus persicae* and the predominance of two ‘superclones’ in Victoria, Australia. Molecular Ecology 12: 3493–3504. 14629364 10.1046/j.1365-294x.2003.01998.x

[8] Chen Y, Vanlerberghe-Masutti F, Wilson LJ, Barchia I, McLoon MO, et al (2013) Evidence of superclones in Australian cotton aphid *Aphis gossypii* Glover (Aphididae: Hemiptera). Pest management science 69: 938–948. 23292942 10.1002/ps.3455

[9] Harrison JS, Mondor EB (2011) Evidence for an Invasive Aphid “Superclone”: Extremely low genetic diversity in Oleander Aphid (*Aphis nerii*) populations in the Southern United States. PLoS ONE 6: e17524. 21408073 10.1371/journal.pone.0017524 PMC3052316

[10] Llewellyn KS, Loxdale HD, Harrington R, Brookes CP, Clark SJ, et al (2003) Migration and genetic structure of the grain aphid (*Sitobion avenae*) in Britain related to climate and clonal fluctuation as revealed using microsatellites. Molecular Ecology 12: 21–34. 12492875 10.1046/j.1365-294x.2003.01703.x

[11] Piffaretti J, Clamens AL, Vanlerberghe-Masutti F, Gupta RK, Call E, et al (2013) Regular or covert sex defines two lineages and worldwide superclones within the leaf curl plum aphid (*Brachycaudus helichrysi*, Kaltenbach). Molecular Ecology 22: 3916–3932. 23786407 10.1111/mec.12371

[12] Facon B, Genton BJ, Shykoff J, Jarne P, Estoup A, et al (2006) A general eco-evolutionary framework for understanding bioinvasions. Trends in Ecology & Evolution 21: 130–135. 16701488 10.1016/j.tree.2005.10.012

[13] Ward S, Gaskin J, Wilson L (2008) Ecological genetics of plant invasion: what do we know? Invasive Plant Science and Management 1: 98–109.

[14] Loxdale HD (2008) Was Dan Janzen (1977) right about aphid clones being a ‘super-organism’, ie a single ‘evolutionary individual’? New insights from the use of molecular marker systems. Mitteilungen der Deutschen Gesellschaft für Allgemeine und Angewandte Entomologie 16: 437–449.

[15] Loxdale HD (2009) What's in a clone: the rapid evolution of aphid asexual lineages in relation to geography, host plant adaptation and resistance to pesticides. In: Schön I, Martens K, van Dijk P, editors. Lost sex. The evolutionary biology of parthenogenesis: Springer Netherlands. 535–557.

[16] Blackman RL, Eastop VF (2000) Aphids of the world crops: an identification and information guide 2nd edition. Chichester, UK: John Wiley & Sons Ltd.

[17] Mead FW (1978) *Melanaphis sacchari* (Zehntner) – Florida – New continental United States record. Cooperative Plant Pest Report 3.

[18] White WH, Reagan TE, Hall DG (2001) *Melanaphis sacchari* (Homoptera: Aphididae), a sugarcane pest new to Louisiana. Florida Entomologist 84: 435–436.

[19] Singh BU, Padmaja PG, Seetharama N (2004) Biology and management of the sugarcane aphid, *Melanaphis sacchari* (Zehntner) (Homoptera: Aphididae), in sorghum: a review. Crop Protection 23: 739–755.

[20] Blackman RL, Eastop VF, Brown PA (1990) The biology and taxonomy of the aphids transmitting barley yellow dwarf virus. In: Burnett PA, editor. World Perspectives on Barley Yellow Dwarf International Workshop. Udine (Italy): CIMMYT. 197–214.

[21] Remaudière G, Remaudière M (1997) Catalogue of the world's Aphididae. Paris, France: INRA.

[22] Blackman RL, Eastop VF (2006) Aphids on the world's herbaceous plants and shrubs. Chichester, UK: John Wiley & Sons Ltd.

[23] Rott P, Mirkov TE, Schenck S, Girard JC (2008) Recent advances in research on *Sugarcane yellow leaf virus*, the causal agent of sugarcane yellow leaf. Sugar Cane International 26: 18–27.

[24] Schenck S, Lehrer AT (2000) Factors affecting the transmission and spread of *Sugarcane yellow leaf virus* . Plant Disease 84: 1085–1088. 10.1094/PDIS.2000.84.10.1085 30831898

[25] Gonçalves MC, Pinto LR, Souza SC, Landell MGA (2012) Virus diseases of sugarcane. A constant challenge to sugarcane breeding in Brazil. Functional Plant Science and Biotechnology 6: 108–116.

[26] Lehrer A, Wu K, Komor E (2009) Impact of *Sugarcane yellow leaf virus* on growth and sugar yield of sugarcane. Journal of General Plant Pathology 75: 288–296.

[27] Rassaby L, Girard JC, Letourmy P, Chaume J, Irey MS, et al (2003) Impact of *Sugarcane yellow leaf virus* on sugarcane yield and juice quality in Réunion Island. European Journal of Plant Pathology 109: 459–466.

[28] Akbar W, Showler AT, Beuzelin JM, Reagan TE, Gravois KA (2011) Evaluation of aphid resistance among sugarcane cultivars in Louisiana. Annals of the Entomological Society of America 104: 699–704.

[29] Fartek B, Nibouche S, Turpin P, Costet L, Reynaud B (2012) Resistance to *Melanaphis sacchari* in the sugarcane cultivar R 365. Entomologia Experimentalis et Applicata 144: 270–278.

[30] Wang F, Zhao S, Han Y, Shao Y, Dong Z, et al (2013) Efficient and fine mapping of RMES1 conferring resistance to sorghum aphid *Melanaphis sacchari* . Molecular Breeding 31: 777–784.

[31] Dogimont C, Bendahmane A, Chovelon V, Boissot N (2010) Host plant resistance to aphids in cultivated crops: genetic and molecular bases, and interactions with aphid populations. Comptes Rendus Biologies 333: 566–573. 20541167 10.1016/j.crvi.2010.04.003

[32] Caillaud CM, Dedryver CA, Di Pietro JP, Simon JC, Fima F, et al (1995) Clonal variability in the response of *Sitobion avenae* (Homoptera: Aphididae) to resistant and susceptible wheat. Bulletin of Entomological Research 85: 189–195.

[33] Lombaert E, Carletto J, Piotte C, Fauvergue X, Lecoq H, et al (2009) Response of the melon aphid, *Aphis gossypii*, to host-plant resistance: evidence for high adaptive potential despite low genetic variability. Entomologia Experimentalis et Applicata 133: 46–56.

[34] Sunnucks P, Hales D (1996) Numerous transposed sequences of mitochondrial cytochrome oxidase I–II in aphids of the genus *Sitobion* (Hemiptera: Aphididae). Molecular Biology and Evolution 13: 510–524. 8742640 10.1093/oxfordjournals.molbev.a025612

[35] Molecular Ecology Resources Primer Development Consortium, Andris M, Aradottir GI, Arnau G, Audzijonyte A, et al (2010) Permanent Genetic Resources added to Molecular Ecology Resources Database 1 June 2010 – 31 July 2010. Molecular Ecology Resources 10: 1106–1108. 21565125 10.1111/j.1755-0998.2010.02916.x

[36] Folmer O, Black M, Hoeh W, Lutz R, Vrijenhoek R (1994) DNA primers for amplification of mitochondrial cytochrome c oxidase subunit I from diverse metazoan invertebrates. Molecular marine biology and biotechnology 3: 294–299. 7881515

[37] Kim H, Lee S (2008) A molecular phylogeny of the tribe Aphidini (Insecta: Hemiptera: Aphididae) based on the mitochondrial tRNA/COII, 12S/16S and the nuclear EF1α; genes. Systematic Entomology 33: 711–721.

[38] Van Oosterhout C, Hutchinson WF, Wills DPM, Shipley S (2004) Micro-Checker: software for identifying and correcting genotyping errors in microsatellite data. Molecular Ecology Notes 4: 535–538.

[39] Arnaud-Haond S, Belkhir K (2007) GENCLONE: a computer program to analyse genotypic data, test for clonality and describe spatial clonal organization. Molecular Ecology Notes 7: 15–17.

[40] Arnaud-Haond S, Duarte C, Alberto F, Serrão E (2007) Standardizing methods to address clonality in population studies. Molecular Ecology 16: 5115–5139. 17944846 10.1111/j.1365-294X.2007.03535.x

[41] Teacher AG, Griffiths DJ (2011) HapStar: automated haplotype network layout and visualization. Molecular Ecology Resources 11: 151–153. 21429113 10.1111/j.1755-0998.2010.02890.x

[42] Dorken ME, Eckert CG (2001) Severely reduced sexual reproduction in northern populations of a clonal plant, *Decodon verticillatus* (Lythraceae). Journal of Ecology 89: 339–350.

[43] Biomatters (2012) Geneious version 5.6.6.

[44] Rebijith KB, Asokan R, Kumar NKK, Krishna V, Chaitanya BN, et al (2013) DNA barcoding and elucidation of cryptic aphid species (Hemiptera: Aphididae) in India. Bulletin of Entomological Research 103: 601–610. 23680306 10.1017/S0007485313000278

[45] Tamura K, Stecher G, Peterson D, Filipski A, Kumar S (2013) MEGA6: Molecular Evolutionary Genetics Analysis Version 6.0. Molecular Biology and Evolution 30: 2725–2729. 24132122 10.1093/molbev/mst197 PMC3840312

[46] Nei M, Kumar S (2000). Molecular evolution and phylogenetics. Oxford University Press, New York.

[47] Raymond M, Rousset F (2004) GenePop on the Web. v: 3.4.

[48] Coeur d'Acier A, Jousselin E, Martin J-F, Rasplus J-Y (2007) Phylogeny of the Genus *Aphis* Linnaeus, 1758 (Homoptera: Aphididae) inferred from mitochondrial DNA sequences. Molecular Phylogenetics and Evolution 42 598–611. 17113793 10.1016/j.ympev.2006.10.006

[49] Kim H, Lee S (2008) Molecular systematics of the genus *Megoura* (Hemiptera: Aphididae) using mitochondrial and nuclear DNA sequences. Molecules and Cells 25: 510–522. 18460900

[50] Foottit RG, Maw HEL, Von Dohlen CD, Hebert PDN (2008) Species identification of aphids (Insecta: Hemiptera: Aphididae) through DNA barcodes. Molecular Ecology Resources 8: 1189–1201. 21586006 10.1111/j.1755-0998.2008.02297.x

[51] Lee W, Kim H, Lim J, Choi HR, Kim Y, et al (2010) Barcoding aphids (Hemiptera: Aphididae) of the Korean Peninsula: updating the global data set. Molecular Ecology Resources 11: 32–37. 10.1111/j.1755-0998.2010.02877.x 21429098

[52] Carletto J, Lombaert E, Chavigny P, Brévault T, Lapchin L, et al (2009) Ecological specialization of the aphid *Aphis gossypii* Glover on cultivated host plants. Molecular Ecology 18: 2198–2212. 19635073 10.1111/j.1365-294X.2009.04190.x

[53] Razmjou J, Vorburger C, Moharramipour S, Mirhoseini SZ, Fathipour Y (2010) Host associated differentiation and evidence for sexual reproduction in Iranian populations of the cotton aphid, *Aphis gossypii* . Entomologia Experimentalis et Applicata 134: 191–199.

[54] Thomas S, Boissot N, Vanlerberghe-Masutti F (2012) What do spring migrants reveal about sex and host selection in the melon aphid? BMC Evolutionary Biology 12: 47. 22471629 10.1186/1471-2148-12-47 PMC3368726

[55] Figueroa CC, Simon JC, Le Gallic JF, Prunier-Leterme N, Briones LM, et al (2005) Genetic structure and clonal diversity of an introduced pest in Chile, the cereal aphid *Sitobion avenae* . Heredity 95: 24–33. 15931255 10.1038/sj.hdy.6800662

[56] Zepeda-Paulo FA, Simon JC, Ramírez CC, Fuentes-Contreras E, Margaritopoulos JT, et al (2010) The invasion route for an insect pest species: the tobacco aphid in the New World. Molecular Ecology 19: 4738–4752. 20958814 10.1111/j.1365-294X.2010.04857.x

[57] Foottit RG, Maw HEL, Pike KS, Messing RH (2012) Aphids (Hemiptera: Aphididae and Adelgidae) of Hawai'i: Annotated List and Key to Species of an Adventive Fauna. Pacific Science 66: 1–30.

[58] Zimmerman EC (1948) Insects of Hawaii. Vol. 5. Homoptera: Sternorrhyncha. Honolulu: University of Hawaii Press. 464 p.

[59] Hall DG (1987) The sugarcane aphid, *Melanaphis sacchari* (Zehntner), in Florida. Journal of the American Society Sugar Cane Technologists 7: 26–29.

[60] Hall DG (1988) Insects and mites associated with sugarcane in Florida. Florida Entomologist 71: 138–150.

[61] Mondor E, Tremblay M, Messing R (2007) Morphological and ecological traits promoting aphid colonization of the Hawaiian Islands. Biological Invasions 9: 87–100.

