# Supplementary figures and images for: Low Genetic Diversity in Melanaphis sacchari Aphid Populations at the Worldwide Scale
Source: PLoS One. 2014 Aug 22;9(8):e106067. doi: 10.1371/journal.pone.0106067 (PMC4141858; doi:10.1371/journal.pone.0106067)

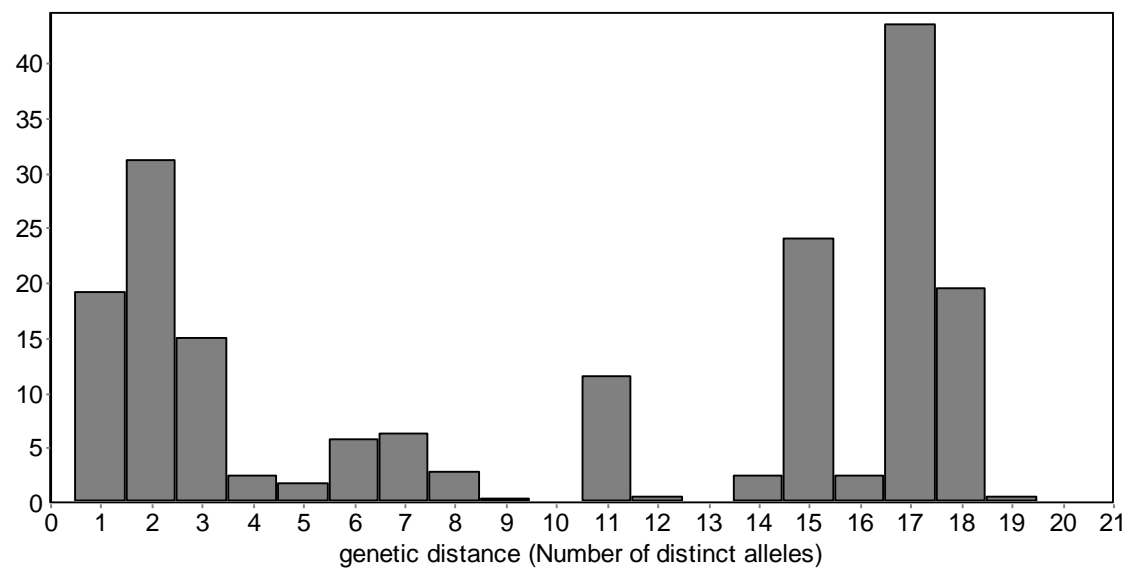

**Figure S1.** Distribution of the pairwise number of different alleles between MLGs.

Supplement: Figure S1 — Distribution of the pairwise number of different alleles between MLGs. (PDF) [file pone.0106067.s001.pdf]
