# Supplementary material for: Low Genetic Diversity in Melanaphis sacchari Aphid Populations at the Worldwide Scale
Source: PLoS One. 2014 Aug 22;9(8):e106067. doi: 10.1371/journal.pone.0106067 (PMC4141858; doi:10.1371/journal.pone.0106067)
Supplement: Figure S2 — Molecular Phylogenetic analysis by Maximum Likelihood method with bootstrap support (10,000 replicates) using 658 bp cytochrome c oxidase subunit I sequences from 100 Melanaphis spp. individuals. (PDF) [file pone.0106067.s002.pdf]

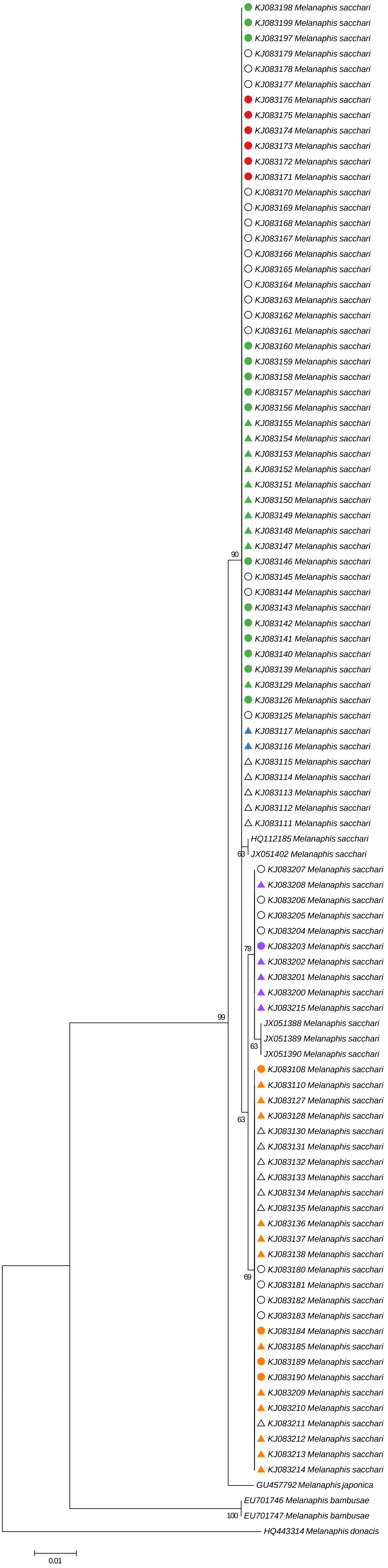


**Figure S2.** Molecular Phylogenetic analysis by Maximum Likelihood method with bootstrap support (10,000 replicates) using 658 bp cytochrome c oxidase subunit I sequences from 100 *Melanaphis* spp. individuals. The evolutionary history was inferred with MEGA6 by using the Maximum Likelihood method based on the General Time Reversible model with gamma distribution of evolutionary rates among sites. The shape of symbols refers to the host plant: sugarcane (triangle) or sorghum (circle). The colour of the symbol refers to the Multilocus Lineage (MLL): A (green), B (pale blue), C (yellow), D (violin), or E (red). Empty symbols mean that microsatellite data were not available.
