# Supplementary material for: Low Genetic Diversity in Melanaphis sacchari Aphid Populations at the Worldwide Scale
Source: PLoS One. 2014 Aug 22;9(8):e106067. doi: 10.1371/journal.pone.0106067 (PMC4141858; doi:10.1371/journal.pone.0106067)
Supplement: Table S2 — Genetic differentiation between MLLs: pairwise FST and significance of the G test computed with GENEPOP. (PDF) [file pone.0106067.s004.pdf]

Table S2. Genetic differentiation between MLLs: pairwise F_{ST} (below diagonal) and significance of the G test (above diagonal) computed with GENEPOP.

	A	B	C	D	E
A		< 0.0001	< 0.0001	< 0.0001	< 0.0001
B	0.586		< 0.0001	< 0.0001	< 0.0001
C	0.6487	0.262		< 0.0001	< 0.0001
D	0.6627	0.341	0.347		< 0.0001
E	0.5336	0.592	0.659	0.694	
