# Supplementary material for: Low Genetic Diversity in Melanaphis sacchari Aphid Populations at the Worldwide Scale
Source: PLoS One. 2014 Aug 22;9(8):e106067. doi: 10.1371/journal.pone.0106067 (PMC4141858; doi:10.1371/journal.pone.0106067)
Supplement: Table S3 — Population genetics parameters for each MLL. (PDF) [file pone.0106067.s005.pdf]

Table S3. Population genetics parameters for each MLL: number of individuals (N), number of MLG, clonal richness (R_{MLG}), probability of departure from Hardy-Weinberg equilibrium (P_{HWE}), probability of heterozygote excess P_{EXC}, probability of heterozygote deficit (P_{DEF}), fixation index (F_{IS}).

MLL	N	MLG	R_{MLG}	P_{HWE}	P_{EXC}	P_{DEF}	F_{IS}
A	39	20	0.500	< 0.0001	< 0.0001	1	-0.736
B	11	4	0.300	< 0.0001	< 0.0001	1	-0.890
C	34	8	0.212	< 0.0001	< 0.0001	1	-0.823
D	12	2	0.091	<0.0001	<0.0001	1	-0.973
E	2	2	1.000	na	na	na	na
